# Characterization of *Sporidiobolus ruineniae* A45.2 Cultivated in Tannin Substrate for Use as a Potential Multifunctional Probiotic Yeast in Aquaculture

**DOI:** 10.3390/jof6040378

**Published:** 2020-12-18

**Authors:** Apinun Kanpiengjai, Chartchai Khanongnuch, Saisamorn Lumyong, Aksarakorn Kummasook, Suwapat Kittibunchakul

**Affiliations:** 1 Division of Biochemistry and Biochemical Technology, Department of Chemistry, Faculty of Science, Chiang Mai University, Chiang Mai 50200, Thailand; 2 Research Center of Microbial Diversity and Sustainable Utilization, Faculty of Science, Chiang Mai University, Chiang Mai 50200, Thailand; 3 Division of Biotechnology, Faculty of Agro-Industry, Chiang Mai University, Mae-Hia, Chiang Mai 50100, Thailand; chartchai.k@cmu.ac.th; 4 Division of Microbiology, Department of Biology, Faculty of Science, Chiang Mai University, Chiang Mai 50200, Thailand; scboi009@gmail.com; 5 Academy of Science, The Royal Society of Thailand, Sanam Suea Pa, Dusit, Bangkok 10300, Thailand; 6 Unit of Excellence in Infectious Disease, Department of Medical Technology, School of Allied Health Sciences, University of Phayao, Muang, Phayao 56000, Thailand; aksarakorn.ku@up.ac.th; 7 Institute of Nutrition, Mahidol University, 999 Phutthamonthon 4 Rd., Nakhon Pathom 73170, Thailand; suwapatkt@gmail.com

**Keywords:** feed additive, probiotic, yeast, *Sporidiobolus ruineniae*, tannase

## Abstract

At present, few yeast species have been evaluated for their beneficial capabilities as probiotics. *Sporidiobolus ruineniae* A45.2, a carotenoid-producing yeast, was able to co-produce cell-associated tannase (CAT), gallic acid and viable cells with antioxidant activity when grown in a tannic acid substrate. The aim of this research study was to identify the potential uses of *S. ruineniae* A45.2 obtained from a co-production system as a potential feed additive for aquaculture. *S. ruineniae* A45.2 and its CAT displayed high tolerance in pH 2.0, pepsin, bile salts and pancreatin. Furthermore, its viable cells were characterized by moderate hydrophobicity, high auto-aggregation and moderate co-aggregation with *Staphylococcus aureus*, *Salmonella* ser. Thyphimurium and *Streptococcus agalactiae*. These attributes promoted *S. ruineniae* A45.2 as a multifunctional probiotic yeast. In addition, the intact cells possessed antioxidant activities in a 100–150 μg gallic acid equivalent (GAE)/mL culture. Remarkably, the fermentation broth demonstrated higher antioxidant activity of 9.2 ± 1.8, 9.0 ± 0.9, and 9.8 ± 0.7 mg GAE/mL culture after FRAP, DPPH and ABTS assays, respectively. Furthermore, higher antimicrobial activity was observed against *Bacillus cereus*, *Staphylococcus aureus* and *Strep. agalactiae*. Therefore, cultivation of *S. ruineniae* A45.2 with a tannic acid substrate displayed significant potential as an effective multifunctional feed additive.

## 1. Introduction

Aquaculture is the most rapidly growing sector of food production throughout the world. Its global demand continues to rise as it is applied to farming practices in ever-increasing proportions [1]. Industrial-scale aquaculture production is recognized as a challenge within the farming sector as it requires sustainable and efficient technologies [2] that address concerns of potential human exposure to microbial diseases and the possibility of severe economic losses across the industry [3]. The use of antibiotics is a simple approach in the control of diseases, however, excess antibiotics that are discharged into the environment are known to be responsible for the spread of antibiotic-resistant genes of pathogenic and commensal bacteria, all of which can lead to increases in drug resistance among animal and human populations. The addition of additives to antibiotics, vaccines, immunostimulants, prebiotics and probiotics, in particular, is an environmentally friendly alternative and recognized as a sustainable strategy [2]. The Food and Agriculture Organization (FAO)/World Health Organization (WHO) defined probiotics as live microorganisms that, when administered in adequate amounts, confer a health benefit upon the host [4]. In terms of their potential applications in aquaculture, yeasts are considered a second leading source of probiotics after bacteria. However, the use of probiotic yeasts is not as popular as bacteria. Indicative of a greater potential for profit than bacterial probiotics, yeasts are not affected by antibacterial compounds and are known to contain various immunostimulant compounds [3,5]. However, their applicable use among a wide variety of animals is limited due to the fact that the normal body temperature of animals is higher than the temperature for the optimal growth of yeast. To date, two yeast species, namely *Saccharomyces cerevisiae* and *Debaryomyces hansenii*, are widely recognized as potential probiotic yeasts [5]. Additionally, yeasts isolated from fish microbiota exhibit certain probiotic properties. These yeast species include *Candida deformans*, *Rhodotorula mucilaginosa*, *Yarrowia lipolytica*, *Metschnikowia viticola* and *Cryptococcus laurentii* [3].

*Sporidiobolus ruineniae* A45.2, isolated from fermented tea-leaves of northern Thailand, namely Miang, is a pigment-producing and tannin-tolerant type of yeast [6]. It is among the range of yeasts commonly found in the intestines of humans [7]. Based on evidence established by previous studies [6,8], *S. ruineniae* A45.2 is assumed to have a unique cell wall structure that serves its tannin-tolerance and may promote the organism as a potential probiotic yeast. On the other hand, its carotenoid pigment is considered highly valuable in terms of the enhancement of some aquaculture pigmentations [9]. Moreover, *S. ruineniae* A45.2 is capable of producing thermostable and pH-stable cell-associated tannase (CAT) and can degrade tannic acid to gallic acid [10]. Tannase is a feed additive enzyme that plays an important role in the reduction of tannins, an antinutritional factor in animal feed. The enzymatic degradation of tannins releases gallic acid that can be used as both an antimicrobial and an antioxidant agent. In aquaculture feed ingredients, tannins come from plant-derived, alternate fish feed ingredients that are used as protein sources, such as soybean meal, rapeseed meal, pea seed meal and mustard oil cake [11]. This drawback leads to significantly decreased levels of cumulative feed intake and digestibility [12]. However, this can be overcome by the addition of tannase.

In previous studies involving the co-production of gallic acid and CAT derived from tannins, both cells and the culture broth rich in gallic acid may be used as a potential source of tannase and gallic acid in the feed industry, respectively. The aims of this research study were to evaluate *S. ruineniae* A45.2 for its potential to be used as a probiotic in aquaculture. Our objectives were to also investigate the potential for fermented broth cultivated in a tannic acid substrate to be further applied as a multifunctional feed additive. Further carotenoids produced by this yeast were also characterized.

## 2. Materials and Methods

### 2.1. Chemicals

Bile salts, 40× pancreatin, pepsin, methyl gallate, gallic acid, rhodanine, 2,4,6-Tris (2-pyridyl)-*s*-triazine (TPTZ), 2,2-diphenyl-1-picrylhydrazyl (DPPH), 2,2′-azino-bis (3-ethylbenzothi-azoline-6-sulfonic acid (ABTS) and potassium persulfate were all of analytic grade and of the highest quality available from Sigma and Sigma-Aldrich (St. Louis, MO. USA). All media used in this research study, including yeast–malt extract broth (YMB), nutrient broth (NB), trypticase soy broth (TSB) and agar, were purchased from HiMedia (Nashik, India).

### 2.2. Microorganisms and Culture Conditions

*S. ruineniae* A45.2 was maintained on yeast-malt extract agar (YMA) at 4 °C for further use. To prepare the seed inoculum, a single colony of yeast was inoculated in YMB and incubated at 30 °C on a 150 rpm rotary shaker for 15–18 h or until the optical density at 600 nm reached 2.0–3.0. *Escherichia coli* TISTR 527, *Salmonella* ser. Thyphimurium TISTR 1472, *Staphylococcus aureus* TISTR 746 and *Bacillus cereus* TISTR 747 were maintained on nutrient agar (NA) and were grown in a nutrient broth (NB) at 37 °C on a 100 rpm rotary shaker when necessary. Furthermore, *Listeria monocytogenes* DMST 17303 and *Streptococcus agalactiae* DMST 11366 were maintained on trypticase soy agar (TSA). These pathogenic bacteria were grown in TSB at 37 °C on a 100 rpm rotary shaker when necessary.

### 2.3. Co-Production of Gallic Acid, CAT and Viable Cells of S. ruineniae A45.2

A total of 10% (*v/v*) of the prepared seed inoculum was transferred into a 1 L stirred tank fermenter (B.E. Marubishi Co. Ltd., Tokyo, Japan) with a 60% working volume of the optimized medium [10] that contained 12.3 g/L tannic acid, 6.91 g/L glucose, 10 g/L yeast extract, 2 g/L (NH_4_)_2_SO_4_, 0.5 g/L tween 80 and 1 g/L glutamate (pH 6.0). Culture conditions were administered at 30 °C with an aeration rate of 1 vvm and an agitation speed of 250 rpm. After 48 h of cultivation, the cells were harvested by centrifugation at 8000 rpm, 4 °C for 10 min and washed twice with phosphate buffer saline (PBS) supplemented with 0.1% (*v/v*) triton X-100. The cell pellets were then resuspended in 0.85% (*w/v*) NaCl to obtain a concentration of 10^8^ cells/mL for further experimentation. 

### 2.4. Tolerance of S. ruineniae A45.2 and Stability of Tannase at Low pH Values

A total of 0.5 mL of the prepared cell suspension (10^8^ cells/mL) was transferred to a 125 mL Erlenmeyer flask containing 49.5 mL of 0.85% (*w/v*) NaCl adjusted to pH 2.0 and 3.0 by 0.1 N HCl. The cell suspension in PBS was then used as a control. All mixtures were incubated at 30 °C for 4 h. Samples were periodically taken for measurement of viable cells by plate count technique and residual tannase activity. Initial cell concentration and tannase activity without incubation were set to 100%. 

### 2.5. Tolerance of S. ruineniae A45.2 and Stability of Tannase in Simulated Gastric Juice

A total of 0.5 mL of the prepared cell suspension (10^8^ cells/mL) was transferred to a 125 mL Erlenmeyer flask containing 49.5 mL of simulated gastric juice (0.3% (*w/v*) pepsin, 0.85% (*w/v*) NaCl, pH 2.0). Cell suspension in PBS was used as a control. All mixtures were incubated at 30 °C for 4 h. Samples were periodically taken for measurement of viable cells by plate count technique and to determine residual tannase activity. Initial cell concentration and tannase activity without incubation was set to 100%. 

### 2.6. Bile Salt Tolerance of S. ruineniae A45.2 and Stability of Tannase 

A total of 0.5 mL of the prepared cell suspension (10^8^ cells/mL) was transferred to a 125 mL Erlenmeyer flask containing 49.5 mL of solution that consisted of 0.85% (*w/v*) NaCl and 0.3% (*w/v*) bile salts. Cells suspended with PBS were used as a control. All mixtures were incubated at 30 °C for 6 h. Samples were periodically taken for measurement of viable cells by the plate count technique to determine residual tannase activity. Initial cell concentration and tannase activity without incubation were set to 100%. 

### 2.7. Tolerance of S. ruineniae A45.2 and Stability of Tannase in Simulated Intestinal Fluid

A total of 0.5 mL of the prepared cell suspension (10^8^ cells/mL) was transferred to a 125 mL Erlenmeyer flask containing 49.5 mL of simulated intestinal fluid (0.3% (*w/v*) bile salts, 0.3% (*w/v*) pancreatin and 0.85% (*w/v*) NaCl). Cells suspended with PBS were used as a control. All mixtures were incubated at 30 °C for 8 h. Samples were periodically taken for measurement of viable cells by plate count technique to determine residual tannase activity. Initial cell concentration and tannase activity without incubation were set to 100%. 

### 2.8. Assay of Tannase

Tannase activity was assayed using the previously described method [13]. Briefly, 50 μL of enzyme solution was mixed with 50 μL of 12.5 mM methyl gallate in 100 mM citrate–phosphate buffer pH 6.5 and incubated at 30 °C. After the incubation procedure, the reaction was stopped by adding 60 μL of 0.667% (*w/v*) methanolic rhodanine and the mixture was left at room temperature (25 °C) for 5 min. Subsequently, 40 μL of 0.5 M KOH was added to the mixture, which was then left at room temperature for 5 min prior to adding 800 μL of distilled water. Absorbance of the mixture was measured at 520 nm. One unit of tannase was defined as the amount of enzyme that liberated 1 μmol of gallic acid per minute under assay conditions.

### 2.9. Cell Surface Hydrophobicity

Yeast adherence was determined by cell surface hydrophobicity. The cell suspension (3 mL) (A_initial_) was transferred to a glass tube (12 × 100 mm) containing 1 mL of chloroform, agitated using a vortex mixer for 2 min and allowed to stand at room temperature for 30 min. The optical density of the aqueous phase (A_final_) was measured at a wavelength of 600 nm. The hydrophobicity index (HPBI) was calculated using the following equation:

$$\mathrm{HPBI}\;(\%)=(1-\frac{A_{\mathrm{final}}}{A_{\mathrm{initial}}})\times 100$$

### 2.10. Auto-Aggregation Assay

A total of 3 mL of the yeast suspension in PBS (A_initial_) was transferred to a glass tube (12 × 100 mm), vortexed for 10 s and incubated at 30 °C for 2 h. Absorbance of the upper part of the mixture (approximately 1 mL) was measured at 600 nm (A_final_). Auto-aggregation was calculated using the following equation:

$$\mathrm{Auto}-\mathrm{aggregation}(\%)=(1-\frac{A_{\mathrm{final}}}{A_{\mathrm{initial}}})\times 100$$

### 2.11. Co-Aggregation Assay

Equal volumes (1.5 mL) of the yeast suspension (A_yeast_) and pathogenic bacterium (A_pathogen_) were transferred into a glass tube (12 × 100 mm), vortexed for 30 s and incubated at 30 °C for 2 h. Absorbance of the upper part of the mixture (A_mix_) was measured at 600 nm. Co-aggregation was calculated using the following equation:

$$\mathrm{Co}-\mathrm{aggregation}(\%)=(1-\frac{A_{\mathrm{mix}}}{(A_{\mathrm{yeast}}+A_{\mathrm{pathogen}})/2})\times 100$$

### 2.12. Adherence of Bacteria onto Yeast Cells

Adhesion of bacteria onto yeast cells was accomplished by mixing 1 mL of the yeast suspension (10^8^ cells/mL) in PBS and 1 mL of each pathogenic bacteria (10^8^ cells/mL). The specimens were then incubated at 30 °C. After 2 h of incubation, 10 μL of the mixture was smeared onto a microscopic slide for Gram-staining [14]. The Gram-stained slide was then used to visualize the adherence of the bacteria onto the yeast cells under a phase-contrast light microscope. The pathogenic bacteria used in the adherence test were *B. cereus*, *E. coli*, *Sal.* Thyphimurium, *Staph. aureus*, *L. monocytogenes* and *Strep. agalactiae*.

### 2.13. Determination of Antimicrobial Activity

The agar well diffusion method was used to determine antagonistic activity of culture broth obtained from the co-fermentation and fermentation in YMB. Briefly, an overnight culture (approximately 10^6^–10^8^ CFU/mL) of the pathogenic bacteria was swabbed onto an NA plate for *B. cereus*, *E. coli*, *Sal.* Thyphimurium and *Staph. aureus* and a TSA plate for *L. monocytogenes* and *Strep. agalactiae*. The wells were prepared by being punched with a 6 mm diameter sterile cork-borer and were filled with 50 μL of sterile culture broth or 50 μg/mL of chloramphenicol as the positive control. The plates were incubated at 30 °C for 18 h.

### 2.14. Analysis of Carotenoids

The previously obtained cell pellet of *S. ruineniae* A45.2 was lyophilized into a dry cell for carotenoid extraction. The freeze-dried cell (0.25 g) was then placed into a screw-cap glass tube (25 × 150 mm) containing 10 mL of acetone and 30 g of glass beads. Cell disruption was carried out through vigorous mixing for 10 min at room temperature. The mixture was filtered through filter paper to collect the cell extract and then centrifuged at 10,000 rpm for 10 min for the purposes of clarification. To quantify total carotenoids, absorbance of the clear extract was measured at 485 nm. The total carotenoid content of the yeast cells was calculated based on the extinction coefficient $\left(E_{1\;\mathrm{cm}}^{1\%}\right)$ of 2680 and expressed in terms of total carotenoids (μg)/g dry cell weight. To determine the carotenoid composition, individual carotenoids were separated by Mightysil RP-18 GP prepacked column (150 × 2.0 mm; Kanto Chemical Co., Inc., Tokyo, Japan) equilibrated with a solution of methanol/acetonitrile (90:10 *v/v*). The conditions were carried out at 30 °C with a flow rate of 1.0 mL/min. The separated carotenoids were detected using a UV detector at 485 nm. Meanwhile, the evaporated cell extract was resuspended in acetone. This was then spotted on a thin layer chromatography (TLC) Silica gel plate (Silica gel 60 F_254_, Merck Millipore, Germany) and developed in a chamber containing hexane/acetone (70:30 *v/v*). The pigments separated by TLC were recovered and dissolved in acetone for measurement of the wavelength of maximum absorbance $\left(\lambda_{\max}\right)$ using a UV-visible spectrophotometer. 

### 2.15. Assay of Antioxidants

A culture of *S. ruineniae* A45.2 obtained from the co-production system was harvested by centrifugation at 8000 rpm for 10 min. The cell pellet was washed twice with PBS solution, suspended in the same solution and the fermented broth was then collected. Both intact cells and cell-free extract were determined for antioxidant activity using three different methods, including ferric-reducing antioxidant power (FRAP), DPPH free-radical-scavenging activity and ABTS free-radical-scavenging activity.

For the FRAP assay, the FRAP reagent consisted of 300 mM acetate buffer pH 3.6, a solution of 10 mM TPTZ in 40 mM HCl and 20 mM FeCl_3_ at a ratio of 10:1:1 (*v/v/v*). The sample solution (0.10 mL) was mixed thoroughly with 3.40 mL of the FRAP reagent for 30 min prior to measuring the absorbance at 593 nm. A standard curve was prepared using different concentrations of gallic acid. The results were expressed in terms of milligram gallic acid equivalent (GAE)/mL culture.

For the DPPH assay, a sample (0.25 mL) was mixed with freshly prepared 40 ppm methanolic DPPH (2.25 mL) and allowed to stand in the dark at room temperature. A decrease in absorbance at 517 nm was determined after 10 min of the incubation process. The concentration of the sample that produced between 20% and 80% inhibition of the blank absorbance was determined and adapted. Radical scavenging activity was expressed as the concentration of the extract required for reduction of the initial concentration of DPPH by 50% (EC_50_) under specified experimental conditions. DPPH radical scavenging activity was expressed in terms of mg GAE/mL culture.

For the ABTS assay, 0.0384 g of ABTS was prepared in 10 mL of water. Subsequently, 5 mL of the solution was mixed with 88 μL of 140 mM potassium persulfate and adjusted to 25 mL with deionized water in a volumetric flask for further experimentation. A total of 1.75 mL of the ABTS solution was mixed thoroughly with 0.25 mL of the sample and allowed to stand in the dark at room temperature for 10 min. A decrease in absorbance at 734 nm was measured. Radical scavenging activity was expressed as the concentration of the extract required for reduction of the initial concentration of ABTS by 50% (EC_50_) under specified experimental conditions. ABTS radical scavenging activity was expressed in terms of mg GAE/mL culture.

## 3. Results

### 3.1. Survival of Yeast and CAT Stability under Simulated Gastrointestinal Tract (GIT) Conditions

**Figure 1:**
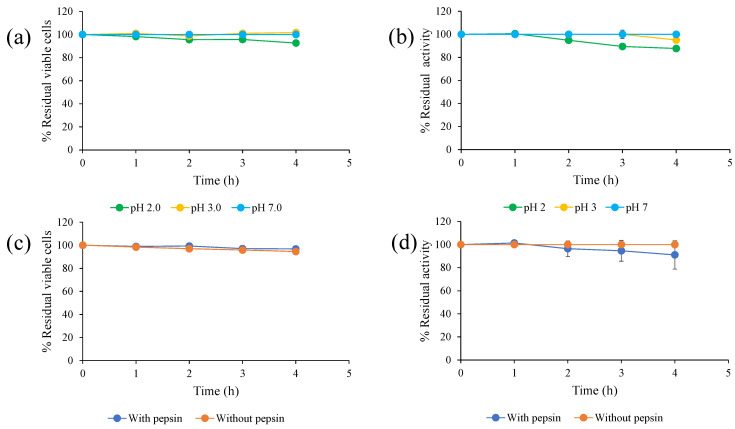
Effect of pH 2.0 and pH 3.0 on (**a**) survival of *S. ruineniae* A45.2 and (**b**) residual CAT activity and effect of pepsin on (**c**) survival of *S. ruineniae* A45.2 and (**d**) residual CAT activity.

Gastric and intestinal conditions were simulated for evaluation of the probiotic properties of *S. ruineniae* A45.2. Under simulated gastric conditions, cell viability of *S. ruineniae* A45.2 and its residual CAT activity were evaluated at pH 2.0 and pH 3.0 (Figure 1a,b) and carried out with and without (Figure 1c,d) the supplementation of pepsin. The results revealed that the values of both pH and pepsin did not significantly affect cell viability of the yeast or its CAT. At a pH value of 2.0, both in the presence and in the absence of pepsin, approximately 90% of the initial viable cells and residual CAT activity were retained. Furthermore, at a pH value of 3.0, the supplementation of pepsin slightly decreased tannase activity but had no effect on cell viability.

**Figure 2:**
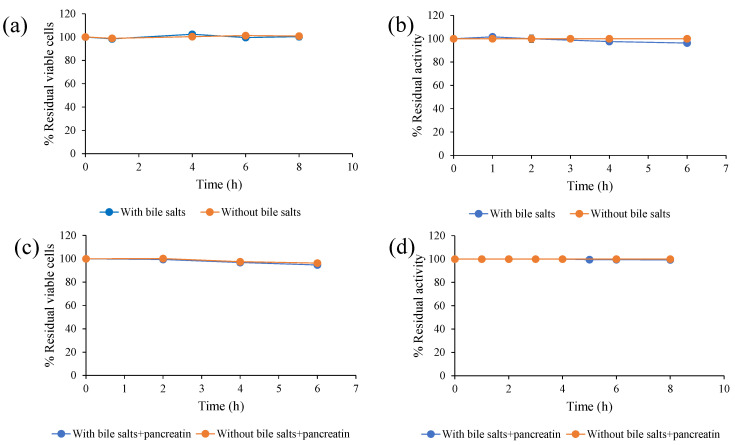
Effect of bile salts on (**a**) survival of *S. ruineniae* A45.2 and (**b**) residual CAT activity and effect of pancreatin in combination with bile salts on (**c**) survival of *S. ruineniae* A45.2 and (**d**) residual CAT activity.

The same results were also obtained when *S. ruineniae* A45.2 and CAT were incubated under simulated intestinal conditions, in which bile salts and pancreatin acted as key factors. It was revealed that *S. ruineniae* A45.2 and CAT were resistant to bile salts (Figure 2a,b) and pancreatin (Figure 2c,d), as they retained 100% residual viable cells and tannase activity after incubation.

### 3.2. Cell Surface Hydrophobicity, Auto-Aggregation and Co-Aggregation

**Figure 3:**
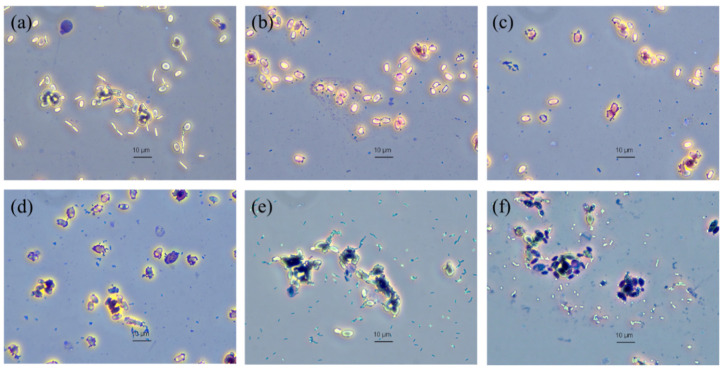
Adherence of (**a**) *B. cereus*, (**b**) *E. coli*, (**c**) *Staph. aureus*, (**d**) *Sal.* Thyphimurium, (**e**) *L. monocytogenes* and (**f**) *Strep. agalactiae* on yeast cell walls observed under a phase-contrast light microscope at 100× magnification.

Cell surface hydrophobicity, auto-aggregation and co-aggregation of *S. ruineniae* A45.2 are presented in Table 1. *S. ruineniae* A45.2 displayed 58.4 ± 2.7% cell surface hydrophobicity. Significantly high values of auto-aggregation were observed at up to 88.2 ± 1.2% along with the ability to be co-aggregated with pathogenic bacteria, including *B. cereus*, *E. coli*, *Staph. aureus*, *Sal.* Thyphimurium, *L. monocytogenes* and *Strep. agalactiae* at different percentages of co-aggregation. *S. ruineniae* A45.2 displayed a stronger co-aggregation ability with *Strep. agalactiae*, *Sal.* Thyphimurium and *Staph. aureus* than other tested pathogenic bacteria. This evidence was in agreement with their adherence ability (Figure 3), wherein pathogenic bacteria obviously adhered to the yeast cells.

### 3.3. Antimicrobial Activity

**Figure 4:**
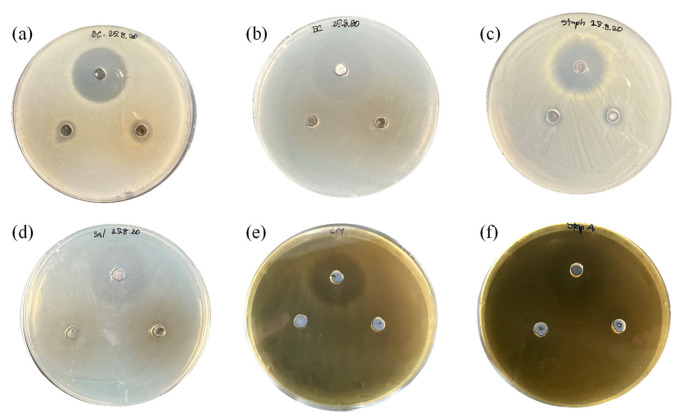
Antimicrobial activity of cell-free extract of *S. ruineniae* A45.2 cultivated in tannic acid substrate at 30 °C for 24 (left) and 48 h (right) of cultivation against (**a**) *B. cereus*, (**b**) *E. coli*, (**c**) *Staph. aureus*, (**d**) *Sal.* Thyphimurium, (**e**) *L. monocytogenes* and (**f**) *Strep. agalactiae* compared to control (top) (50 μg/mL chloramphenicol)

The antagonistic effects of *S. ruineniae* A45.2 were observed after being exposed to various indicator microorganisms, including *B. cereus*, *E. coli*, *Sal.* Thyphimurium, *Staph. aureus*, *L. monocytogenes* and *Strep. agalactiae*. Culturing periods of 24 and 48 h of the co-production system were tested in comparison with specimens cultured in YMB. Only the supernatant obtained from co-production exhibited antimicrobial activity against some pathogenic bacteria (Figure 4), i.e., *B. cereus*, *Staph. aureus* and *Strep. agalactiae*.

### 3.4. Carotenoids Produced by S. ruineniae A45.2

**Figure 5:**
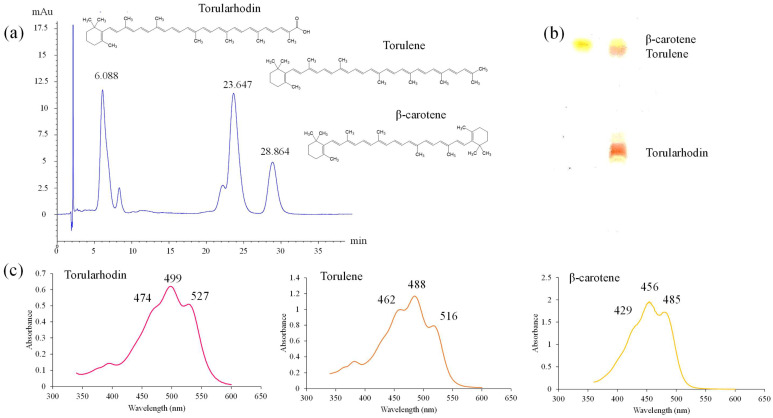
Characterization of pigments produced by *S. ruineniae* A45.2 (**a**) Separation of pigments by HPLC and (**b**) TLC and (**c**) Visible absorption spectra of the major pigments.

*S. ruineniae* A45.2 grown in tannic acid were harvested after 48 h of cultivation, lyophilized and used for carotenoid extraction. Identification and characterization of the carotenoid pigment was performed by HPLC. Three main peaks were separated from the carotenoid extracts (Figure 5a). These peaks corresponded to the three spots that were isolated and visualized by TLC (Figure 5b). The first spot from the bottom was rosy-red in color and migrated with slower mobility than the second spot, which appeared orange to red in color, while the third spot was yellow in color and migrated with the same degree of mobility as β-carotene. These pigments were identified based on their Visible absorbance maxima (Figure 5c). The least degree of polar pigment was identified as β-carotene due to similar absorbance maxima values recorded at 429 nm and 485 nm, with the maximal degree of absorbance recorded at 456 nm. The most notable polar pigment revealed a spectrum with three absorption maxima at wavelengths of 474, 527 with the absorption optimum at 499 nm, thereby identified as torularhodin. The second most polar pigment was torulene with three absorbance maxima values recorded at 462 nm and 516 nm, with an absorption maximum value recorded at 488 nm. Total carotenoids produced by *S. ruineniae* A45.2 after cultivation at 30 °C for 48 h were recorded at 88.0 ± 0.2 μg/g dry cell weight, equivalent to 500 ± 130 μg/L culture.

### 3.5. Antioxidant Activity

Antioxidant activities of intact cells of *S. ruineniae* A45.2 and cell-free extract obtained from the co-production system were measured using FRAP, DPPH and ABTS assays. Overall, there were no significant differences in the antioxidant activities among the different methods. Antioxidant activities ranging from 100–120 μg GAE/mL culture and 110–150 μg GAE/mL culture were detected from the intact cells obtained from 24-h and 48-h periods of cultivation, respectively (Table 2). The cell-free extract revealed significantly higher antioxidant activities than the intact cells. The antioxidant activities of 5.6–6.8 mg GAE/mL culture and 9.0–9.8 mg GAE/mL culture were obtained from the cell-free extract fraction after 24-h and 48-h periods of cultivation, respectively. Activities of both intact cells and cell-free extract increased relative to the incubation time of the co-production system.

## 4. Discussion

In this study, *S. ruineniae* A45.2 and its culture broth obtained from the co-production of cells, gallic acid and tannase were characterized for their potential use in animal feed, specifically in feed prepared for fish and other aquatic organisms. *S. ruineniae* A45.2 was isolated from Miang, which is rich in tannins and considered a microbial inhibitor [6,8]. The cell wall structure and composition of *S. ruineniae* A45.2 are believed to promote its growth along with high concentrations of tannic acid, yet this yeast was found to be a promising probiotic. When used as a functional probiotic yeast, growth temperature is a crucial limitation for the application of probiotics in animals, since the yeast must be able to survive and grow at the animal’s normal body temperature in order to enhance the animal’s growth performance and promote the health of the animal [15]. Typically, the growth temperatures of yeasts range from 0 to 47 °C with an optimal temperature between 25 and 30 °C [16], yet probiotic yeasts might actively function when they are used in aquaculture. Probiotic yeasts are less popular than bacteria but can offer some major physiological contributions over bacteria. These include their cell volume and the production of a wide spectrum of simple and more complex compounds that may be beneficial to the health of aquatic organisms. However, only a few varieties of probiotic yeasts have been isolated for aquaculture applications. It was reported that marine and other aquatic environments, along with the gut microbiota of aquatic organisms, are potential sources of probiotic yeasts. In addition to *S. cerevisiae*, *D. hansenii* is a ubiquitous yeast that is frequently associated with fish and marine environments [7]. As of yet, no reports of using *S. ruineniae* as a probiotic yeast have been identified.

To be a good probiotic yeast, it must be able to successfully survive under gastrointestinal tract (GIT) conditions and provide beneficial conditions for the enhancement of the health of the host. In this study, both *S. ruineniae* A45.2 and its CAT were exposed to GIT conditions in order to assess the degree of residual cell viability and CAT. The temperature used in this research study was 30 °C, as it was identified as an optimal temperature of *S. ruineniae* A45.2 (data not shown). Considering cell viability, *S. ruineniae* A45.2 resisted low pH values ranging from 2.0 to 3.0, which were within the range found in the stomachs of fish. The degree of acidity in the stomach of a fish can vary depending on the fullness of the stomach and the species of the fish [17,18]. Moreover, *S. ruineniae* A45.2 was not found to be affected by the digestive enzymes we tested, namely pepsin and pancreatin (a mixture of amylase, protease and lipase). These attributes are considered important selection criteria for a good probiotic yeast [19]. On the other hand, the CAT of *S. ruineniae* A45.2 exhibited a good degree of thermostability and pH stability. Surprisingly, positive stability values were observed under simulated GIT conditions by retaining more than 90% of initial activity after treatment. This indicates that the yeast species could be applicable in the aquafeed industry. Plant-based products in fish diets contain valuable proteins used to replace fishmeal. These plant feed ingredients contain considerable amounts of tannins that can have an adverse effect on animals by reducing the nutritional value of the feed [11]. This circumstance can also decrease the palatability of the feed due to an unpleasant taste caused by a high concentration of tannins [12]. The results of this study indicate that both cells of *S. ruineniae* A45.2 might be able to survive in transit through the stomach and small intestines and function effectively in the large intestines. However, its CAT might be stable in stomach environments and could be active in the intestines, as the environments are similar to the known optimal values for pH and temperature.

Cell surface hydrophobicity is defined as a nonspecific interaction in adhesion between probiotic microorganisms onto GIT epithelial cells, where they may provide prophylactic and therapeutic benefits [20]. Colonization in the intestinal epithelial cell wall and mucosal surfaces can prevent pathogenic bacteria adhesion and inflammatory reactions [21]. Yet, hydrophobicity is an important attribute for selecting potential probiotics. *S. ruineniae* A45.2 showed high cell surface hydrophobicity toward chloroform and was comparable with those reported in *Bacillus subtilis* [19,21], various strains of *Lactobacillus* sp. [22] and *Sac. unisporus* [20].

Auto-aggregation is defined as aggregation among yeast cells to form flocs and colonize the intestinal environment of the host when the cells approach harmful conditions [20,23]. Probiotic microorganisms should be associated with higher auto-aggregation than pathogenic microorganisms [22], specifically *Strep. agalactiae*, a representative fish pathogen. Under the same experimental conditions as this study, the percentage auto-aggregation of pathogenic bacteria ranged between 15–35% for *L. monocytogenes*, *Sal.* Thyphimurium and *Staph. aureus* [22]. Within 2 h of the auto-aggregation test, *S. ruineniae* reported 88.2 ± 1.2%, which was higher than previously reported probiotic yeasts, namely *P. kluyveri*, *Issatchenkia orientalis*, *P. kudriavzevii* [24], *Yarrowia lipolytica*, *Wickerhamomyces anomalus* and *Sac. cerevisiae* [23]. Auto-aggregation capacity is strain-specific, while a capacity greater than 50% displayed the potential to prevent the invasion of various other pathogenic microorganisms through film formation.

Co-aggregation is defined as the close interaction between probiotics and different pathogenic bacteria [23]. It was reported that adherence of enteric bacteria onto yeast cells is irreversible, thus transient passage of the bacteria occurs through GIT and subsequent flushing out in the feces [20]. The co-aggregation ability of *S. ruineniae* A45.2 agreed with its adherence ability. This could be explained by the specific fimbriae present on bacteria with mannan on yeast cells and the electrostatic and hydrophobic nonspecific bindings [20]. 

No antimicrobial activity of *S. ruineniae* A45.2 against the tested pathogenic bacteria was detected when it was cultivated in YMB. It is therefore implied that no antimicrobial metabolite was produced by the organisms typically identified in various yeasts [20,24,25,26]. Most yeasts scavenge pathogenic infection by indirect mechanisms such as auto-aggregation, co-aggregation and adherence ability [27]. On the contrary, the growing of *S. ruineniae* A45.2 in tannic acid containing medium led to the release of gallic acid, which enhanced the antimicrobial activity of the culture broth against *B. cereus*, *E. coli*, *Staph. aureus* and *Strep. agalactiae*. The results suggest that production of the yeast should be performed in the presence of tannic acid to promote gallic acid production and CAT, thereby gaining antimicrobial activity. Supplementation of gallic acid in animal feed, especially aquatic feed, was scarcely reported. Current research found that the supplementation of gallic acid in broiler diets at levels ranging from 75 to 100 mg/kg improved the performance of broiler chicks in terms of feed utilization, breast muscle yield and oxidative stability, while positively modulating jejunum intestinal morphology [28]. Hence, our results provide supplemental, supportive evidence for the use of gallic acid as an alternative to antibiotics in animal feed or for the determination of synergistic interactions of gallic acid that could enhance the effects of antibiotics.

*S. ruineniae* A45.2 is a basidiomycetous yeast that forms a natural pink-red pigment made up of carotenoids. The pigments extracted from the yeast were separated into three types of carotenoids based on the separation by HPLC and TLC. These pigments displayed distinctively different visible spectra. The most polar pigment showed a rosy-red color and had a similar visible spectrum to torularhodin, while the others displayed a similar spectrum to torulene and β-carotene as the second most polar and the least polar pigments, respectively [29,30,31]. However, structural elucidation of these compounds must be confirmed. Currently, carotenoid-producing yeasts are mainly represented by the genera *Rhodosporidium*, *Xanthophylomyces Rodotorula* and *Sporobomyces*. The latter genus has a close relationship to the genus *Sporidiobolus* and represents the main source of torulene and torularhodin [32]. The quantity of total carotenoids produced by *S. ruineniae* A45.2 was in the range of those produced by the yeast studied in previously published reports [31,33].

After cell wall components, some probiotic yeasts exhibit multifunctional potential in the production of bioactive compounds with certain antioxidant properties, such as carotenoids, organic acids and glutathione [24]. As *S. ruineniae* A45.2 is a carotenoid-producing yeast, it is likely that it possesses antioxidant capacity. The intact cells and cell-free extract obtained from YMB were evaluated for their antioxidant activity (data not shown). No antioxidant activity was detected in the cell-free extract, while the intact cells possessed approximately 10 times lower the degree of antioxidant activity than that obtained from cultivation in tannic acid. This may have resulted from the presence of β-glucan as a component of yeast cell wall composition [34,35]. Cultivation of *S. ruineniae* A45.2 in tannic acid could potentiate the antioxidant activity of not only intact cells but also cell-free extracts. It was determined that the fermentation of *S. ruineniae* A45.2 induced the production of CAT which strongly affected the degradation of tannic acid, resulting in gallic acid production. During the degradation of tannic acid, large amounts of gallic acid were released into the fermentation broth and attached to the yeast cell surface, reported in previous studies [10,36]. The antioxidant activity in terms of gallic acid equivalent is likely a consequence of gallic acid content, as reported in previously published studies [10]. This result agrees with previously reported evidence published on the fermentation of plant-based foods [37], including grape seed flour and extracts [38], as well as Miang [39], as sources of *S. ruineniae* A45.2.

Overall, *S. ruineniae* A45.2 may be capable of exhibiting the beneficial characteristics attributed to a probiotic yeast that can be used for aquaculture. Cultivation of the yeast in tannic acid substrate might provide a number of benefits. These benefits include the assertion that yeast cells can be a source of antioxidant agents, tannase and carotenoids for aquatic organisms. Furthermore, it is believed that the resulting culture broth can display strong antioxidant activity as well as the potential to display antimicrobial activity against some pathogenic bacteria, especially fish pathogens. Therefore, this research study described and verified an alternative integrative strategy for the production of feed additives. To our knowledge, this is the first report to suggest that *S. ruineniae* exhibits probiotic properties.

## 5. Conclusions

*S. ruineniae* A45.2 was tolerant to simulated GIT conditions, displaying tolerance to pH 2.0, pepsin, bile salts and pancreatin. A high percentage of auto-aggregation was observed, and this species co-aggregated various pathogenic bacteria, specifically *Strep. agalactiae*, and adhered to some specific strains of pathogenic bacteria. These are considered beneficial attributes that support the use of *S. ruineniae* as a probiotic yeast. The fermentation of tannic acid to gallic acid has resulted in the co-production of CAT, gallic acid and viable yeast cells. Moreover, CAT was found to be stable and may be able to function under simulated GIT conditions, while the cells possessed antioxidant activity. Thus, *S. ruineniae* A45.2 as a carotenoid- and CAT-producing yeast could be labeled as a multifunctional probiotic yeast suitable for the feed of animals, particularly aquatic animals. In addition, its cell-free extract derived from the co-production system could be a potential alternative source of natural antioxidants and antimicrobial agents. 

## Figures and Tables

**Table 1:** Cell surface hydrophobicity, auto-aggregation and co-aggregation against pathogenic bacteria.

Properties	%
Cell surface hydrophobicity	58.4 ± 2.7
Auto-aggregation	88.2 ± 1.2
Co-aggregation	
*B. cereus*	36.2 ± 2.8
*E. coli*	33.8 ± 0.7
*Staph. aureus*	44.0 ± 0.8
*Sal.* Thyphimurium	45.8 ± 0.1
*L. monocytogenes*	37.8 ± 2.1
*Strep. agalactiae*	51.5 ± 2.6

**Table 2:** FRAP, DPPH and ABTS antioxidant activity of cell-free extract and intact cells obtained from co-production of gallic acid and viable cells of *S. ruineniae* A45.2.

Time (h)	Cell-Free Extract (mg GAE/mL)			Intact Cells (μg GAE/mL)		
	FRAP	DPPH	ABTS	FRAP	DPPH	ABTS
24	5.6 ± 0.8	6.8 ± 0.6	6.4 ± 0.4	122.1 ± 9.3	108.5 ± 2.4	104.7 ± 0.8
48	9.2 ± 1.8 *	9.0 ± 0.9 *	9.8 ± 0.7 *	143.6 ± 3.4 *	114.6 ± 1.4 *	111.1 ± 0.5 *

* significant difference within a column (*p* < 0.05).
